# Radiation exposure reduction in peripheral interventions using digital variance angiography versus conventional angiography

**DOI:** 10.1038/s41598-025-06361-7

**Published:** 2025-07-09

**Authors:** Till Schürmann, Ulrich Beschorner, Dirk Westermann, Thomas Zeller, Fabian Bamberg, Christopher L. Schlett, Thomas Stein, Elias Noory

**Affiliations:** 1 Department of Diagnostic and Interventional Radiology, University Medical Center Freiburg, Faculty of Medicine, University of Freiburg, 79106 Freiburg, Germany; 2 Department of Cardiology and Angiology, University Heart Center, University Medical Center Freiburg, Faculty of Medicine, University of Freiburg, 79189 Bad Krozingen, Germany

**Keywords:** Digital variance angiography, Digital subtraction angiography, Endovascular peripheral interventions, X-ray, Radiation dose, Interventional cardiology, Imaging techniques

## Abstract

Digital variance angiography (DVA) exhibits promising prospects with respect to radiation exposure in digital subtraction angiography (DSA). This study aimed to determine the reduction of radiation dose in endovascular peripheral interventions (EPI) using DVA. The DVA imaging tool v6.0 (Kinepict Medical Imaging Tool, version 6.0.4, Kinepict Health Ltd., Budapest, Hungary) was utilized for patients undergoing EPI using digital angiography. EPI normal dose (EPI-ND) protocols were adapted from 1.20 to 0.81 µGy/frame to EPI low dose (EPI-LD) protocols using DVA-LD acquisitions with 0.36 µGy/frame and occasionally 0.24 µGy/frame based on specific examination requirements. The dose area product (DAP) was evaluated and contrast-to-noise ratio (CNR) was measured for each DSA acquisition. Evaluation included 370 EPI-ND and 62 EPI-LD using DVA-LD of three lower extremity regions (mean age: 73 ± 11 years, 67% male). LD protocols decreased median DAP of ND protocols significantly by 62.0% in pelvic, 53.8% in femoral and popliteal, and 59.4% in cruro-pedal regions, respectively (*p* < .005). DVA-LD increased median CNR significantly compared to DSA-LD (*p* < .001), and was equal to DSA-ND (*p* > .15). Image quality was enhanced by CNR_DVA−LD_/CNR_DSA−ND_ ratio of 1.9 in pelvic, 2.4 in femoral and popliteal and in cruro-pedal regions. DVA reveals significant radiation dose reduction in lower extremity EPIs and enhances image contrast while decreasing noise.

## Introduction

Digital subtraction angiography (DSA)^1,2^ contributes as essential interventional tool to diagnose and treat stenosis and occlusions in endovascular peripheral interventions (EPI). However, interventions apply X-ray radiation that is related to negative deterministic and stochastic health effects for both patients and interventional staff conducting the examination^3–5^.

Digital variance angiography (DVA) is a motion-based X-ray imaging technique^6^which automatically processes the distribution of iodinated contrast medium (ICM) in the vascular system based on the change of contrast and its variance^6^. The technique depicts its benefits in conventional DSA of EPI such as lower limb angiography and angioplasty^7–11^. The contrast agent in motion is significantly enhanced by DVA while studies have shown that the post-processed DSA by DVA was found to exhibit at least twice the signal-to-noise ratio (SNR)^11^ or contrast-to-noise ratio (CNR)^10,12^ using ICM. In parallel, the noise in the background can be reduced. In addition, publications^10,13^ show a significant increase in image quality in subjective evaluations by radiologists and other interventional specialists. An increased image quality in X-ray examinations invariably indicates a savings potential for radiation dose^5,11^. Here, both the savings in radiation dose^7,8^ and ICM^14^ could be confirmed with at least maintained or mostly improved image quality. On the one hand, studies show indicators of significantly enhanced image quality, while on the other hand, radiation exposure or ICM volume can be reduced^7–11,14^.

Despite the growing evidence of the benefits in DVA, the limitation of the above-mentioned studies is the evaluation and comparison exclusively of *summated* DSA^2^ and DVA images. However, in clinical routine, EPIs of lower extremities are typically conducted with a series of individual angiography images where the contrast agent flow is visually tracked by the interventional staff. Therefore, we aimed to demonstrate the impact of DVA compared to series of individual angiographic image acquisitions. In this retrospective, monocentric study, the potential radiation exposure reduction using digital variance angiography (acronym: *REDUNDANCY*) and the difference in image quality is evaluated and compared to conventional EPIs including DSA applications as well as digital unsubtracted angiography (DUA) where background tissue is not subtracted by the raw acquisition data.

## Materials and methods

This study was approved by the Institutional Review Board (Ethics Committee – University of Freiburg; EK 24-1048-S1-retro on 03/12/2024) and carried out in accordance with the Declaration of Helsinki and its later amendments. Written informed consent was obtained. The pseudonymized data was collected from the electronic patient file as well as from the angiographic images.

### Patient selection

We retrospectively included 370 patients from conventional EPIs with normal dose (EPI-ND) protocols between 11/2022 and 11/2023 and 62 patients from EPIs with low dose (EPI-LD) protocols using the DVA (DVA-LD) imaging tool v6.0 (Kinepict Medical Imaging Tool, version 6.0.4, Kinepict Health Ltd., Budapest, Hungary, https://kinepict.com/) between 11/2023 and 01/2024. Patient demographics are shown in Table 1.

**Table 1:**
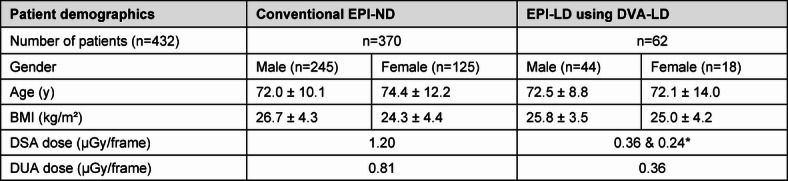
Patient demographics presented with mean ± standard deviation. Clinical standard protocols were changed from conventional EPI-ND (including DSA-ND using 1.20 µGy/frame and DUA-ND using 0.81 µGy/frame) to EPI-LD using DVA-LD (including DSA-LD using 0.36 µGy/frame und 0.24 µGy/frame and DUA-LD using 0.36 µGy/frame).

*LD cohort of DSA was utilized with 0.36 µGy/frame in 84% and 0.24 µGy/frame in 16% based on examination requirements and situational need.

*EPI* Endovascular peripheral intervention, *DSA* digital subtraction angiography, *DVA* digital variance angiography, *DUA* digital unsubtracted angiography, *ND* normal dose, *LD* low dose.

**Fig. 1:**
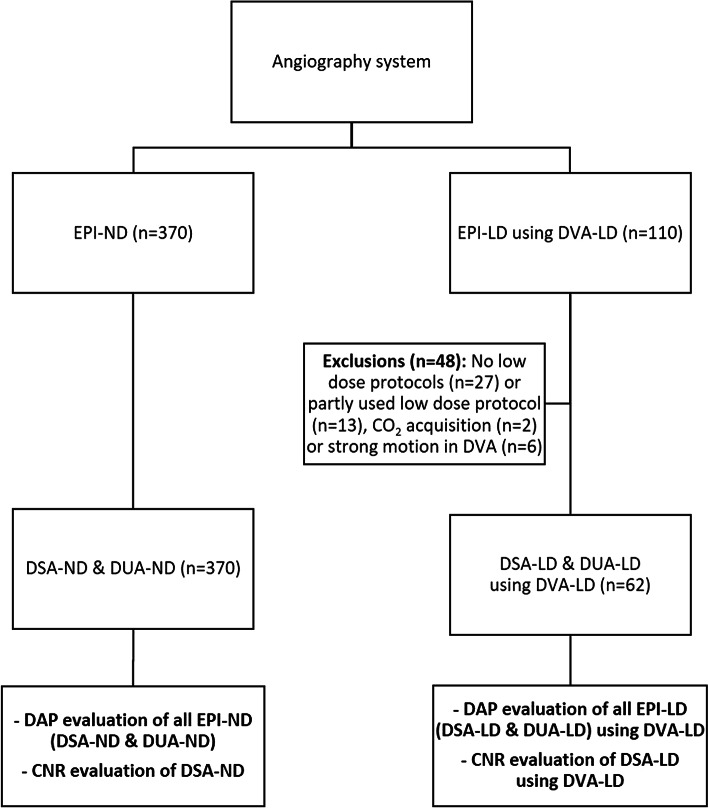
Flowchart of the patient inclusion and exclusion, respectively. Conventional endovascular peripheral interventions (EPIs) with normal dose (EPI-ND) protocols were conducted with 370 patients between 11/2022 and 11/2023 and 62 patients using digital variance angiography (DVA) with low dose (DVA-LD) protocols between 11/2023 and 01/2024. The endpoints encompassed the evaluation of radiation dose using the dose area product (DAP) and image quality with regards to the contrast-to-noise ratio (CNR) in the ND groups compared to the LD groups. While the DAP evaluation included all EPIs using digital subtraction angiography (DSA) and digital unsubtracted angiography (DUA) acquisitions, CNR evaluation was exclusively conducted in DSA acquisitions due the possible comparability.

Figure 1 demonstrates the flow chart of patient selections regarding its image acquisition and evaluation methodology. The EPI cohorts included acquisitions using DSA and furthermore, DUA with visible background tissue. The ND group was treated with the institutional standard radiation dose for the angiographic image acquisition protocols whereas the LD group was treated with 56% (DUA) and 70–80% (DSA) reduced dose per frame (Table 1).

While all patients of the EPI-ND group were retrospectively included in the selection process, 48 of 110 patients of the EPI-LD group were excluded (Fig. 1). First, 27 cases were excluded since within the use of the DVA software, no low dose protocols were applied due to changing staff that was unfamiliar with the application of the DVA software. Second, LD protocols were only partly used with less than 60% of DVA acquisitions due to increased patient motion and ND protocols were applied instead (*n* = 13). Third, CO_2_ acquisitions were not included in the study due to insufficient data (*n* = 2). Lastly, in some cases, strong motion artifacts appeared in the DVA acquisitions (*n* = 6).

### Study design

Patients` data was retrospectively analyzed using our dose management system (DoseM, version VER2040 BN2 (G3), INFINITT Europe GmbH, Frankfurt am Main, Germany, https://infinitteurope.com/en/dose-management-system/) and collected for radiation dose and image quality evaluation. ND and LD examinations were each subdivided into endovascular interventions of the different vascular regions regarding the pelvic, femoral and popliteal, and cruro-pedal region. While the body regions of the EPI-ND group could be automatically assigned with the dose management software, the EPI-LD group was evaluated and subdivided individually by images from our picture archiving and communication system (PACS, DeepUnity Diagnost, version 2.0.2.2, Dedalus HealthCare GmbH, Bonn, Germany, https://www.dedalus.com/dach/de/our-offer/products/deepunity-diagnost/). In the EPI-LD group, the ratio of the individual interventions to each body region was considered and the majority of pelvic, femoral and popliteal, and cruro-pedal image acquisitions were assigned to the respective region. This subdivision is subject to the legal terms of the current national diagnostic reference levels (DRLs) of Germany^15^.

### Image acquisition and change of clinical standards

Images of EPI were acquired using an angiography system (Siemens Artis Zee, Siemens Healthineers, Forchheim, Germany). ICM volume (Ultravist 300; Bayer Vital GmbH, Leverkusen, Germany) was generally utilized with a dilution of 1:1 with saline solution, which was injected by hand as in our clinical routine.

In general, both the DSA and DUA protocols are used for all lower extremity regions using standard ND acquisition and adapted LD acquisitions. However, the standard DUA acquisitions are associated with an inferior image quality in adipose patients where DSA acquisitions are preferred in clinical routine. In respect to the proportional protocol utilization, DSA acquisitions are preferably used for the pelvic and cruro-pedal region while DUA acquisitions are increasingly used for the femoral and popliteal region.

For the ND group, clinical standard protocols using a radiation dose of 1.2 µGy/frame with a frame rate of 6 frames/second for DSA and 0.81 µGy/frame with a frame rate of 7.5 frames/second for DUA were utilized. For the LD group, clinical standards were changed with protocols using 0.36 µGy/frame (in 60% of the cases) as well as 0.24 µGy/frame (in 40% of the cases) using a frame rate of 6 frames/second for DSA and using 0.36 µGy/frame with a frame rate of 4 frames/second for DUA based on specific examination requirements and situational need in clinical routine. In addition to the DSA-LD and DUA-LD acquisitions, static DVA-LD images were acquired by the raw image data of the image series generating the angiography images, which were processed by the integrated DVA software in our angiography system. Here, DVA-LD acquisitions were primary used to diagnose pathologies in the endovascular system.

It must be noted that the image quality of DVA-LD acquisition was not sufficient for diagnosis in occasional cases due to patient motion, where an additional DSA-ND acquisition must be conducted to assess the angiography images.

### Radiation dose and image quality endpoints

Endpoints encompassed the evaluation of the radiation dose and the parallel impact on the image quality (Fig. 1). For the evaluation of the radiation dose, the overall dose area product (DAP) was collected for each patient from the EPI-ND group and EPI-LD group using DVA-LD acquisitions in the pelvic, femoral and popliteal region, and cruro-pedal region, respectively. Here, all image acquisitions including DSA, DUA and fluoroscopy (FL) were processed and a detailed subanalysis to its proportions of the overall DAP was conducted. In contrast, for the evaluation of the image quality, exclusively DSA images were compared to DVA images in each region due to the incomparability of DUA and DVA acquisitions, which is outlined in the discussion section. In order to evaluate the image quality, CNR was calculated by defined rectangular regions of interest (ROIs) on vessels with ICM in relation to the subtracted background tissue and background standard deviation (SD) according to the following equation

$$\:CNR=\:\frac{({MEAN}_{ICM\:}\:-\:{MEAN}_{background})}{{SD}_{background}}$$

while ROIs were equally distributed from large (> 3 mm ⌀) to small (< 1 mm ⌀) vessels using a ROI manager of ImageJ (Fiji, version 2.14.0/1.54, National Institutes of Health, Bethesda, MD, USA, https://imagej.net/ij/index.html).

**Fig. 2:**
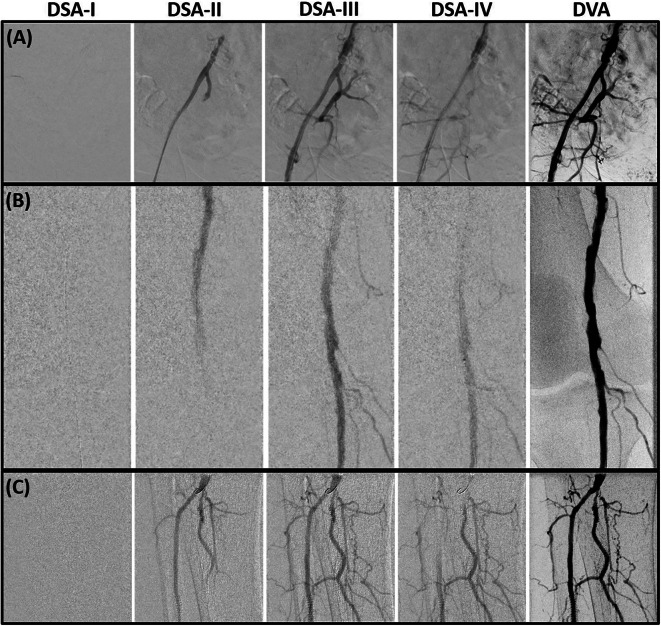
Pelvic (**A**), femoral and popliteal (**B**), and cruro-pedal (**C**) region. The summated digital variance angiography (DVA) image significantly enhances image quality compared to the digital subtraction angiography (DSA) series I–IV (depicting 4 out of 28 (**A**), 48 (**B**) and 28 (**C**) DSA images) of the low dose (LD) acquisitions. Enhancements of iodinated contrast medium (ICM) by DVA depict small vessel structures in detail while the conventional DSA offers a reduced contrast-to-noise ratio (CNR) using LD acquisitions for standalone diagnostic interpretations.

Accordingly, CNR was measured in single DSA-ND and DSA-LD images in the DSA image series as well as in the summated DVA-LD images. In respect of the DSA series, CNR was calculated at the subjective highest contrast of the single DSA image in the acquisition series. The comparison of DSA-LD image series and summated DVA-LD images is demonstrated for instance in Fig. 2 for different lower limb regions. Moreover, the ratio (R value) of each pairwise defined ROI was calculated by CNR_DVA−LD_/CNR_DSA−LD_ in order to exhibit the benefit of DVA in LD protocols.

### Statistical analysis

The collected, study-relevant and pseudonymized data was entered from our PACS and dose management system database while the software MATLAB (MathWorks, version R2024a, Natick, MA, USA, https://de.mathworks.com/products/new_products/release2024a.html) was used for evaluation and statistical analysis. For the analysis of the DAP and CNR, median and interquartile range [IQR] were calculated by the data sets. Significant differences were evaluated by the Mann-Whitney U test for both metrics due to the absence of a Gaussian distribution. An error probability of *p* < .05 is considered significant.

## Results

### Patient cohort

A total of 432 patients (mean age: 72.8 ± 10.9 years, 66.9% male) were retrospectively included between 11/2022 and 01/2024 while the patient groups and its characteristics are presented in Table 1. For both groups, EPI-ND and EPI-LD using DVA-LD, interventions could successfully be conducted in all cases.

### Patient motion

In our study, additional acquisitions using ND protocols in the LD cohort were occasionally necessary and must be repeated up to 8.1% (up to 16.5% in DSA-LD and up to 4.6% in DUA-LD) of overall acquisitions since the motion of specific patients caused severe artifacts in DVA-LD acquisitions, which could not be used for diagnosis. Especially, pelvic angiographic acquisitions needed to be repeated in our study due to increased motion artifacts and resulted in an increased median DAP for DUA-LD using DVA-LD compared to DUA-ND acquisitions (Fig. 4). In this case, image quality evaluations such as the CNR were not conducted since the image series was not applicable. Nevertheless, the additional acquisitions using ND protocols are included in the following radiation dose analysis of the DVA-LD cohort.

### Radiation dose analysis

**Fig. 3:**
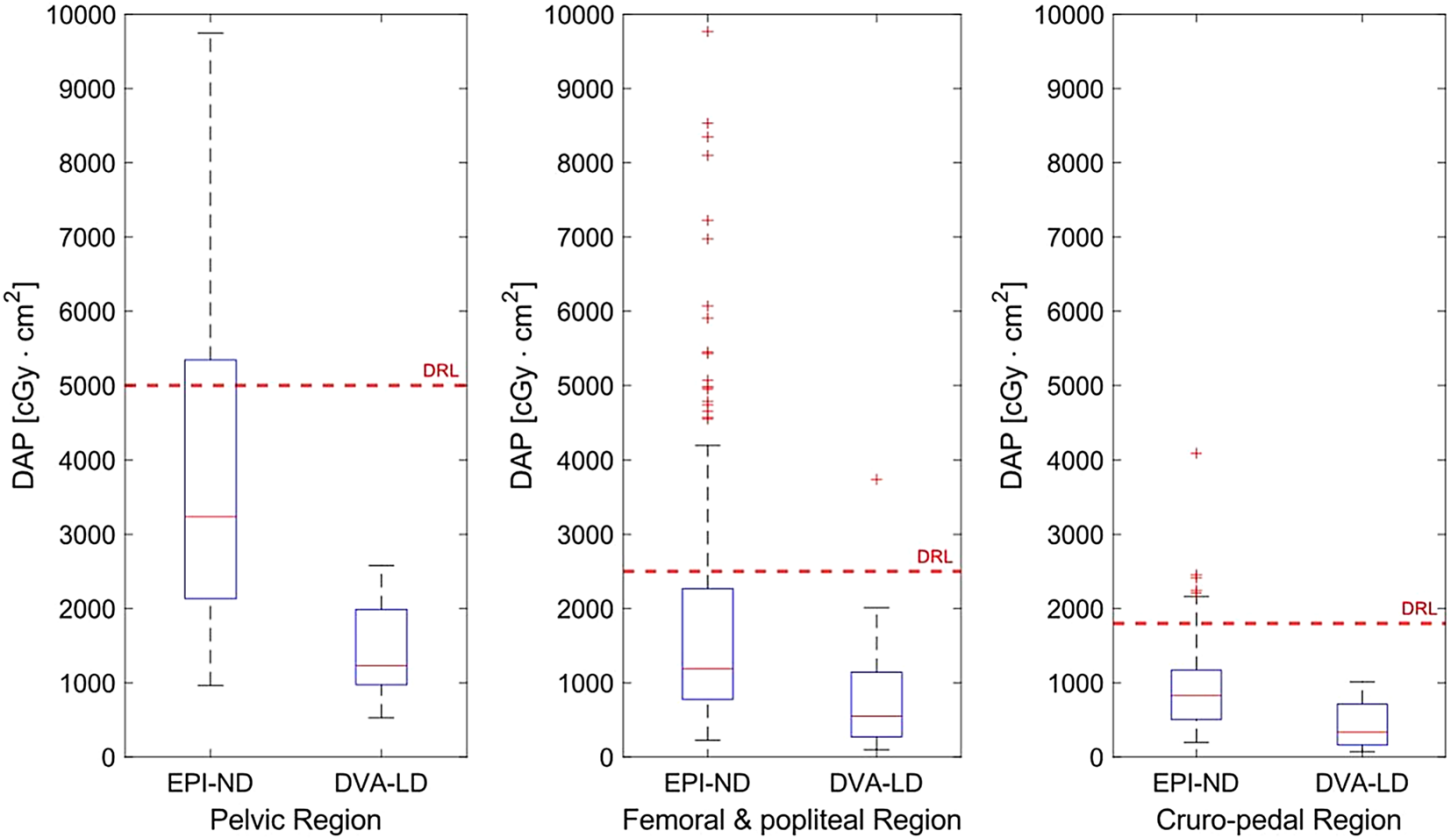
Dose area product (DAP) of endovascular peripheral interventions with low dose (EPI-LD) using digital variance angiography with low dose (DVA-LD) imaging decreases risk of exceeding national diagnostic reference levels (DRLs) at 5000, 2500 and 1800 cGy·cm² for pelvic, femoral and popliteal, and cruro-pedal regions, respectively. Median DAP of EPI with normal dose (EPI-ND) is significantly higher compared to DVA-LD in each region, respectively (*p* < .005).

Overall DAP of EPI-ND (including DSA-ND and DUA-ND) for each patient, respectively, was significantly decreased by EPI-LD (including DSA-ND and DUA-LD) protocols using DVA-LD with median [and IQR] from 3238.6 [3011.4] cGy·cm² to 1230.4 [1013.2] cGy·cm² in pelvic regions, from 1190.9 [1491.7] cGy·cm² to 550.8 [872.2] cGy·cm² in femoral and popliteal regions, and from 827.6 [666.2] cGy·cm² to 336.0 [552.0] cGy·cm² in cruro-pedal regions by 62.0%, 53.8%, and 59.4%, respectively (*p* < .005) (Table 2). Results of the DAP are illustrated in Fig. 3 while DRLs of Germany are defined and depicted for 5000, 2500 and 1800 cGy·cm² in the pelvic, femoral and popliteal, and cruro-pedal region, respectively.

**Table 2:**
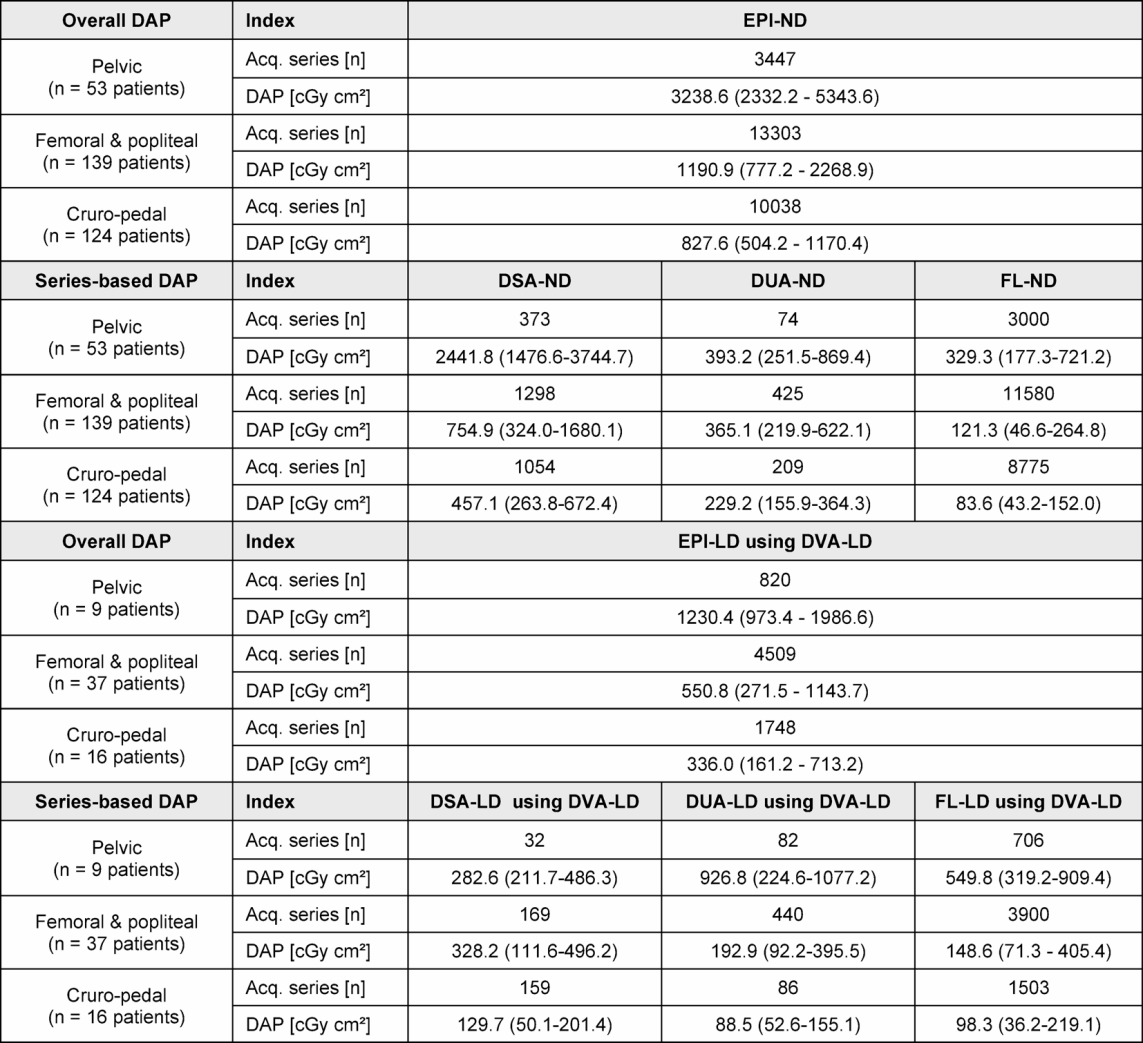
Medi﻿an and interquartile range (Q1–Q3) of series-based distribution analysis for different acquisition protocols of overall DAP per patient for the pelvic, femoral and popliteal, and cruro-pedal region, respectively.

*EPI* endovascular peripheral intervention, *DSA* digital subtraction angiography, *DVA* digital variance angiography, *DUA* digital unsubtracted angiography, *FL* fluoroscopy, *ND* normal dose, *LD* low dose, *DAP* dose area product.

**Fig. 4:**
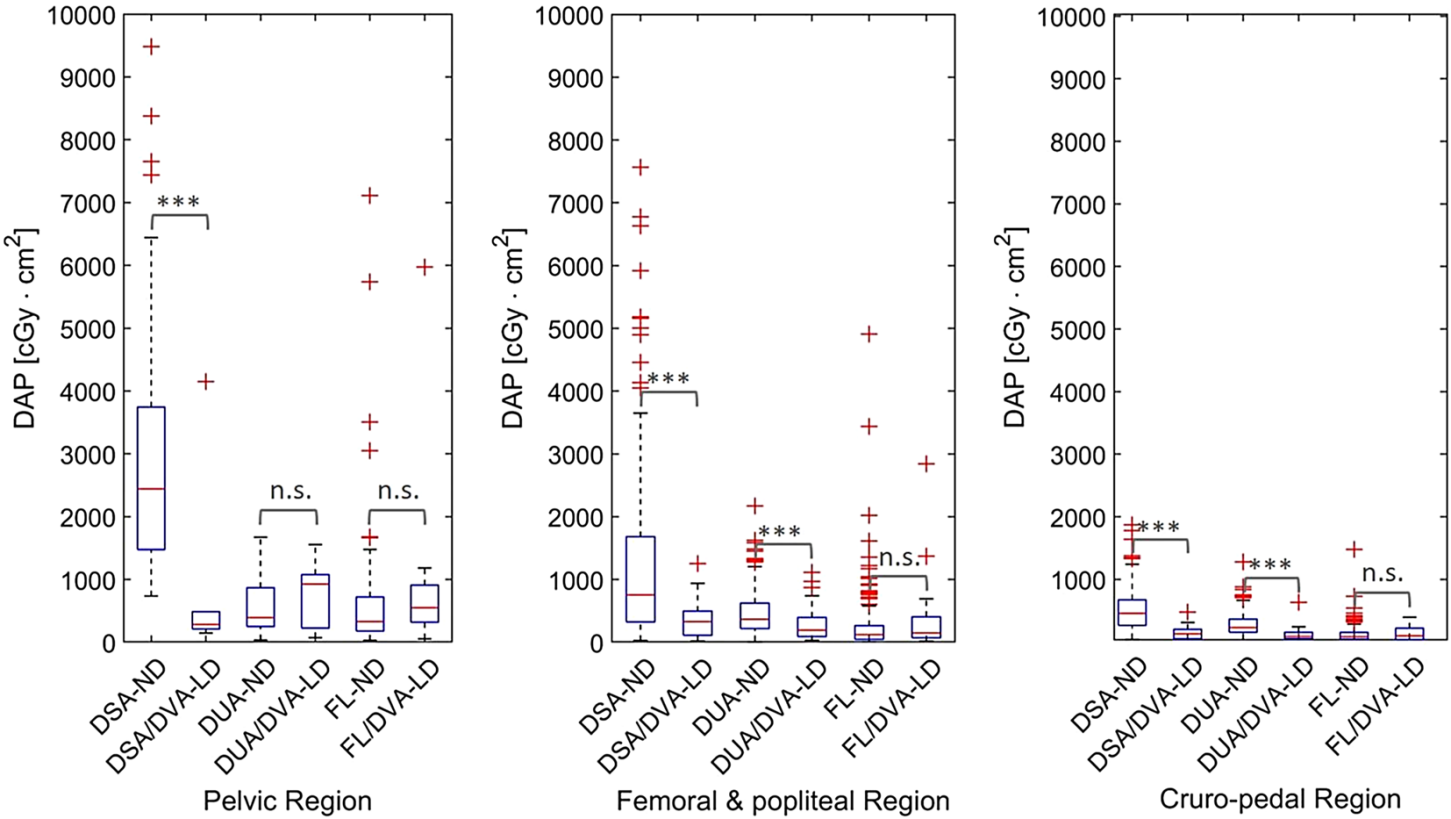
Series-based distribution analysis of overall DAP for different acquisition protocols that were extracted from all EPI-ND and EPI-LD interventions, respectively. Mann-Whitney U test was used for statistical analysis while significant differences are indicated (***p < .005 & n.s. *p* > .05). *EPI* Endovascular peripheral intervention, *DSA* digital subtraction angiography, *DVA* digital variance angiography, *DUA* digital unsubtracted angiography, *FL* fluoroscopy, *ND* normal dose, *LD* low dose, *DAP* dose area product.

In parallel to the overall radiation dose, the median DAP of DSA-ND acquisitions in Fig. 4 and Table 2 were significantly decreased with DSA-LD acquisitions using DVA-LD by 88.4%, 56.5%, 71.6% in each region, respectively (*p* < .005), while the DUA-LD acquisitions were significantly decreased by 47.2% and 61.4% in the femoral and popliteal, and in the cruro-pedal region, respectively (*p* < .005). However, the median DAP of the DUA-ND acquisitions were increased with the DUA-LD acquisitions using DVA-LD by 57.6% in the pelvic region showing no significant differences (*p* > .05). Furthermore, differences of DAP values between ND and LD fluoroscopy acquisitions were not significant (*p* > .05).

### Image quality analysis

Considering the DSA-LD acquisitions with reduced radiation dose in Fig. 2 for instance, DVA-LD images exhibit substantially enhanced ICM contrast and decreased background noise in all regions.

**Fig. 5:**
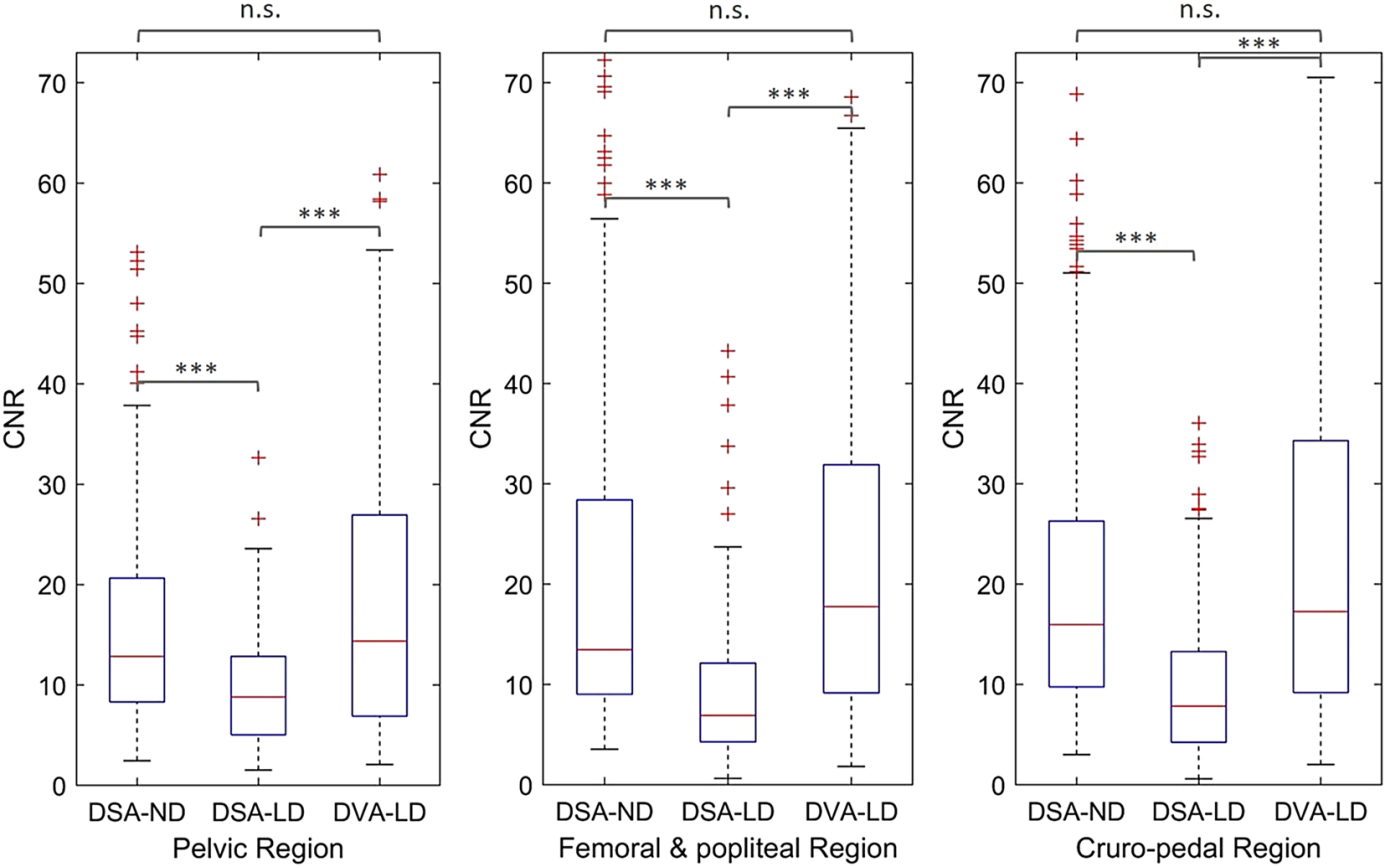
Enhancements of iodinated contrast medium (ICM) by digital variance angiography with low dose (DVA-LD) depict significantly increased contrast-to-noise ratio (CNR) in comparison to conventional digital subtraction angiography with low dose (DSA-LD) acquisitions (***p < .001). CNR differences of DVA-LD compared to DSA with normal dose (DSA-ND) acquisitions were not significant (n.s.).

With a total of 1340 ROIs (312 pelvic, 536 femoral and popliteal, 492 cruro-pedal) in DSA-ND acquisitions and 1644 ROIs (316 pelvic, 708 femoral and popliteal, 620 cruro-pedal) in DSA-LD and DVA-LD acquisitions, respectively, CNR calculation is summarized in Table 3. Median [and IQR] of CNR in DSA-LD increased significantly by DVA-LD from 8.8 [7.9] to 14.4 [20.0] in pelvic regions (*p* < .001), from 6.9 [7.8] to 17.8 [22.7] in femoral and popliteal regions (*p* < .001), and from 7.8 [9.0] to 17.3 [25.1] in cruro-pedal regions (*p* < .001) (Fig. 5). The enhancement in CNR is verified by the R value (CNR_DVA−LD_/CNR_DSA−LD_ ratio) with 1.9 [1.3] in pelvic regions, with 2.4 [1.3] in femoral and popliteal regions, and with 2.4 [1.5] in cruro-pedal regions. The median CNR of DVA-LD was also higher compared to DSA-ND images although interventions were conducted with reduced radiation dose (Fig. 5). However, the Mann-Whitney U test showed no significant differences in pelvic (*p* = .49), femoral and popliteal (*p* = .59), and cruro-pedal (*p* = .15) regions, respectively.

**Table 3:** Median and interquartile range (Q1–Q3) of CNR for DSA-ND and DSA-LD using DVA-LD protocols as well as R values for the pelvic, femoral and popliteal, and cruro-pedal region.

Region	*n* of ROIs (ND)	CNR DSA-ND	*n* of ROIs (LD)	CNR DSA-LD	CNR DVA-LD	*R* values
Pelvic	312	12.8 (8.3–20.7)	316	8.8 (5.0–12.9)	14.4 (6.9–26.9)	1.9 (1.4–2.7)
Femoral & popliteal	536	13.5 (9.0–28.4)	708	6.9 (4.3–12.1)	17.8 (9.2–31.9)	2.4 (1.9–3.2)
Cruro-pedal	492	16.0 (9.8–26.3)	620	7.8 (4.3–13.3)	17.3 (9.2–34.3)	2.4 (1.8–3.3)

Mann-Whitney U test showed significant differences regarding CNR comparison of DSA-ND vs. DSA-LD and DSA-LD vs. DVA-LD (*p* < .001). CNR differences of DSA-ND vs. DVA-LD were not significant (*p* = .49, *p* = .59, *p* = .15 in pelvic, femoral and popliteal, and cruro-pedal region, respectively).

*R values describe the CNR ratio of DVA-LD/DSA-ND.

*CNR* contrast-to-noise ratio, *DSA* digital subtraction angiography, *DVA* digital variance angiography, *DUA* digital unsubtracted angiography, *ND* normal dose, *LD* low dose.

## Discussion

DVA exhibits promising prospects with respect to radiation exposure and ICM in DSA. This study aimed to determine the reduction of radiation dose in EPI using series of individual image acquisitions of DSA and DUA acquisitions instead of the summated DSA.

### Collection of data

In respect to the DVA-LD cohort, 62 cases were the maximum number of conducted interventions using the DVA software apart from the exclusions. A substantially larger number of EPI-ND interventions could be collected, since these were still archived automatically with the dose management system, which matched the cases to the pelvic, femoral and popliteal, and cruro-pedal region. Since a larger number of data increases the stability and validity of the statistical analysis, an increased dataset of the EPI-ND cohort of 370 cases was used, while the DVA-LD cohort was limited to 62 cases.

### Methodology of image acquisition and situational needs

DVA is an imaging technique, which processes the raw image data series of individual EPI images and summarizes these in one contrast enhanced summated image with the entire vessel structure. Therefore, studies in the past^7,8,10,11,14^ compared the enhancement of summated DVA images with summated DSA images, conventionally and typically processed by the image summation of the early- and late-phase^2^in order to compare equal imaging visualization. However, a series of individual images as DSA series, which are not summarized, is typically used in clinical routine. This concerns also our institution where the conventional DSA or DUA series are utilized for EPIs in our medical center. Therefore, individual DSA images such as in Fig. 2 are compared at the subjective highest contrast to the summated DVA images using the CNR evaluation as described in the method section. With the additional DVA software tool, the imaging technique enables a further static image allowing to detect vessels with stenosis, which could possibly not be noticed with the decreased ICM contrast in the conventional DSA series. Nevertheless, the DSA-LD acquisition series additionally provides flow information of the contrast agent despite the reduced CNR of the images. Although the contrast agent of DSA-LD images is more difficult to visualize especially in small vessels, the flow information still supports the detection of stenosis. Accordingly, the combination of static DVA images and additional DSA-LD acquisition series can improve the diagnosis of peripheral artery diseases.

Moreover, it must be noted that our institution generally used relatively high frame rates in regards to the DSA and DUA acquisitions, which were applied in accordance with the recommended presets of the manufacturer as standard protocols. However, the current state-of-the-art present standardized frame rates of less than two frames/second for the lower extremity regions^16^. Yet, the parallel radiation dose reduction from 1.20 µGy/frame to 0.36 µGy/frame was already proved by past studies and the functionality and robustness of the DVA software using a lower frame rate of two frames/second in the pelvic and one frames/ second in the femoral and popliteal as well as in the cruro-pedal region could be confirmed^7,8^.

In this study, LD protocols (0.24 or 0.36 µGy/frame) were utilized whenever the operator anticipated that DVA processing would reliably maintain diagnostic image quality. Conversely, ND protocols (1.20 or 0.81 µGy/frame) were switched in situational needs, for instance in specific procedural circumstances in which the LD image quality might be insufficient for standalone diagnostic interpretation. However, our general diagnostic assessment was based on the combined evaluation of the available DSA-LD and DVA-LD acquisitions. This integrative approach enabled an adequate vessel visualization and diagnostic evaluation for the operator.

The situational needs included complex procedures or critical steps when crossing tight, heavily calcified, or chronic total occlusions, especially if multiple devices such as catheter exchanges were required, operators preferred ND protocols to ensure optimal visualization of guidewire movement and lesion boundaries. Furthermore, motion of the target vessel (e.g. severe patient discomfort, inability to remain still, or frequent breathing/abdominal motion in pelvic imaging) can obscure the vascular structures with DVA’s post-processing in some cases. In such scenarios, repeating an acquisition at ND was often faster and more reliable for clinical decision-making. Moreover, during the early adoption phase, if the operator or supporting staff were not sufficiently comfortable adjusting parameters for DVA-LD acquisitions, they reverted to the established ND protocol to avoid disrupting workflow.

These situational needs typically arose in the minority of cases (overall in 16.5% in DSA-LD and 4.6% in DUA-LD procedures). Whenever possible and feasible, LD protocols with DVA were used to achieve maximal radiation dose reduction. However, our priority remained robust to ensure procedural success and patient safety by use of unambiguous imaging of the vascular pathology.

Lastly, it must be noted that DVA images were connected to an additional delay time of 1–3 s due to the increased computational power needed in contrast to the acquisition of DSA series.

### Patient motion

As described in the results, patient motion can be a crucial disadvantage in DVA imaging due to severe artifacts. Dynamic images of conventional DSA (or DUA) acquisitions with its flowing contrast agent are reproduced as static DVA image of the overall dynamically flowing contrast agent. Figure 2 shows how the dynamic flow of the contrast agent from DSA-I to DSA-IV is processed to one static DVA image with the overall contrast agent flow. Since DVA processes the dynamic DSA series based on every motion in the image series, artifacts of patient motion occur when the background tissue is moving and is processed by the DVA software. In regards to the radiation dose reduction per frame, not each acquisition involved a low dose protocol with 0.24 or 0.36 µGy/frame in the EPI-LD cohort. This was the case when background tissue was in motion. Here, ND protocols were utilized in proportion up to 16.5% with 1.2 µGy/frame in the DSA-LD or up to 4.6% with 0.81 µGy/frame in the DUA-LD cohort in respect to the overall acquisitions where DVA was assumed not to be beneficial. Furthermore, EPIs were generally excluded in the LD group if the majority was related to ND acquisitions (Fig. 1).

### Variability of ICM injection

Since a retrospective study was conducted, we could not instruct every operator to inject the same amount of contrast agent as they would for conventional DSA and some of the operators tested the potential reduction of ICM in rare cases. Furthermore, the contrast agent dilution was occasionally adapted to the specific indication or complexity and patient characteristics such as cardiac output, ejection fraction, stenosis or occlusion of the affected arteries, and the in- and outflow tract. Therefore, ICM was occasionally reduced from a 1:1 up to 1:5 dilution of selected EPIs in order to check the functionality of DVA in the ICM reduction. These infrequent acquisitions were also included in the radiation dose and image quality analysis meaning that the CNR of DSA-LD and DVA-LD acquisitions may be underestimated in this study.

The variability of operators can include aspects such as variable injection volumes, different dilution ratios beyond the standard 1:1 and variations in vascular access, which may introduce statistical heterogeneity into our analysis. However, the overall magnitude and consistency of the observed radiation dose reduction and the improved CNR with DVA demonstrate that these variations did not compromise the main findings.

Since the median CNR of DVA-LD is still superior to DSA-ND images, the parallel reduction in radiation dose and ICM is potentially feasible, which shall be evaluated in further studies. This potential is also implied by a study in the past where the CNR of DVA-LD acquisitions were significantly higher than in DSA-ND acquisitions regarding radiation dose reduction^8^. In particular, this can be beneficial in DUA acquisitions since the additional subtraction image of a DSA image is not needed for the DVA processing.

### Limitations of image quality evaluation

Since DVA acquires only subtracted images, patient groups of ND and LD acquisitions were subdivided into DSA with image subtraction for the CNR analysis and into DUA without image subtraction of the background, in which no CNR analysis was conducted. However, both angiographic image acquisitions were included in the radiation dose analysis for all EPIs. This methodology was applied since the CNR evaluation of DVA is exclusively comparable with DSA-ND and DSA-LD acquisitions due to the equal image visualization in contrast to DUA acquisitions. Nevertheless, the beneficial image quality impact of DVA regarding the CNR of the DSA acquisitions can also be transferred on DUA acquisitions since the image processing technique of DVA is equal for both acquisition modes. Here, the shifting variance is calculated by the raw image data for all acquisition modes^6^. This transfer and a sufficient CNR could be achieved in the DUA acquisitions since a lower radiation dose reduction of the DUA group was conducted in order to use doses per frames with 0.36 µGy/frame, which are at least as high as in the DSA acquisitions. This procedure assured that the CNR of the DUA-LD acquisitions is at least on the same level as the DSA-ND acquisitions.

Furthermore, it must be noted that a subjective qualitative image quality evaluation was not conducted in this study but it was assessed objectively by the CNR. Subjective ratings of the image quality would also be a beneficial determinant for future analysis, especially in prospective studies where categories can be controlled more strictly.

## Conclusions

DVA reveals significant radiation dose reduction in endovascular peripheral interventions of lower extremities and significantly enhances image contrast in terms of ICM while decreasing noise. Accordingly, DVA also ensures adequate clinical image quality in LD image acquisitions. In parallel, DVA can significantly reduce (stochastic) health risk effects of radiation exposure for both patients and staff performing endovascular peripheral interventions.

## Data Availability

The datasets generated during and/or analyzed during the current study are not publicly available, but are available from the corresponding author on reasonable request.

## Abbreviations

DSA: Digital subtraction angiography
DVA: Digital variance angiography
EPI: Endovascular peripheral intervention
DUA: Digital unsubtracted angiography
FL: Fluoroscopy
ICM: Iodinated contrast medium
ND: Normal dose
LD: Low dose
DAP: Dose area product
SNR: Signal-to-noise ratio
CNR: Contrast-to-noise ratio

## Acronym

REDUNDANCY: Radiation Exposure reDUctioN using Digital vAriaNCe angiographY
